# First translational consensus on terminology and definitions of colonic motility in animals and humans studied by manometric and other techniques

**DOI:** 10.1038/s41575-019-0167-1

**Published:** 2019-07-11

**Authors:** Maura Corsetti, Marcello Costa, Gabrio Bassotti, Adil E. Bharucha, Osvaldo Borrelli, Phil Dinning, Carlo Di Lorenzo, Jan D. Huizinga, Marcel Jimenez, Satish Rao, Robin Spiller, Nick J. Spencer, Roger Lentle, Jasper Pannemans, Alexander Thys, Marc Benninga, Jan Tack

**Affiliations:** 1 NIHR Nottingham Biomedical Research Centre (BRC), Nottingham University Hospitals NHS Trust and the University of Nottingham, Nottingham, UK; 2 Nottingham Digestive Diseases Centre, School of Medicine, University of Nottingham, Nottingham, UK; 3 Human Physiology and Centre of Neuroscience, College of Medicine, Flinders University, Bedford Park, South Australia Australia; 4 Department of Medicine, University of Perugia Medical School, Perugia, Italy; 5 Division of Gastroenterology and Hepatology, Mayo Clinic, Rochester, MN USA; 6 Department of Paediatric Gastroenterology, Great Ormond Street Hospital for Sick Children, London, UK; 7 Department of Gastroenterology and Surgery, Flinders Medical Centre, Adelaide, South Australia Australia; 8 Department of Pediatric Gastroenterology, Nationwide Children’s Hospital, The Ohio State University, Columbus, OH USA; 9 Department of Medicine, Farncombe Family Digestive Health Research Institute, McMaster University, Hamilton, Ontario Canada; 10 Department of Cell Physiology, Physiology and Immunology and Neuroscience Institute, Universitat Autònoma de Barcelona, Barcelona, Spain; 11 Division of Gastroenterology/Hepatology, Augusta University, Augusta, GA USA; 12 Discipline of Human Physiology, School of Medicine, Flinders University, Bedford Park, South Australia Australia; 13 Digestive Biomechanics Group, College of Health, Massey University, Palmerston North, New Zealand; 14 Department of Paediatric Gastroenterology and Nutrition, Emma Children’s Hospital/Academic Medical Centre, Amsterdam, Netherlands; 15 Translational Research Center for Gastrointestinal disorders (TARGID), Department of Clinical and Experimental Medicine, University of Leuven, Leuven, Belgium

**Keywords:** Gastrointestinal system, Enteric nervous system, Gastrointestinal models

## Abstract

Alterations in colonic motility are implicated in the pathophysiology of bowel disorders, but high-resolution manometry of human colonic motor function has revealed that our knowledge of normal motor patterns is limited. Furthermore, various terminologies and definitions have been used to describe colonic motor patterns in children, adults and animals. An example is the distinction between the high-amplitude propagating contractions in humans and giant contractions in animals. Harmonized terminology and definitions are required that are applicable to the study of colonic motility performed by basic scientists and clinicians, as well as adult and paediatric gastroenterologists. As clinical studies increasingly require adequate animal models to develop and test new therapies, there is a need for rational use of terminology to describe those motor patterns that are equivalent between animals and humans. This Consensus Statement provides the first harmonized interpretation of commonly used terminology to describe colonic motor function and delineates possible similarities between motor patterns observed in animal models and humans in vitro (ex vivo) and in vivo. The consolidated terminology can be an impetus for new research that will considerably improve our understanding of colonic motor function and will facilitate the development and testing of new therapies for colonic motility disorders.

## Abstract (web summary)

This Consensus Statement provides a conceptual and methodological framework to expand research on colonic motility in experimental animals and humans. The work is intended to facilitate the development of new drugs for common colonic motility disorders and of appropriate diagnostic and therapeutic algorithms for the management of paediatric and adult patients.

## Introduction

The human colon performs several essential functions including fermentation of nutrients, absorption of water and electrolytes, and storage and timely expulsion of faecal contents^1^. These functions require a complex, highly integrated and regulated activity of the colonic muscle that is critical for maintaining health and quality of life.

Alterations in colonic motor function have been implicated in the genesis of symptoms associated with various bowel motility disorders, such as functional diarrhoea or constipation and IBS^2,3^. The study of colonic motility using colonic manometry has been recommended by guidelines and expert reviews to identify colonic motor dysfunction in treatment-refractory constipation in children and adults before consideration of surgical intervention^3–5^.

There is ample agreement that the fundamental nature of the cellular mechanisms that regulate colonic motor activity is well preserved across mammalian species, including humans, despite the diversity in anatomical features of the colon, and the consumption of a variety of diets. However, the introduction of high-resolution manometry (HRM) to study human colonic motor function in the past 5 years has revealed that our current concepts of normal colonic motor activity are somewhat limited and might even be flawed^6–8^. This problem is further complicated by the fact that, over the years, different techniques and protocols have been applied to study colonic function^9^.

Thus, a consensus conference was organized in Leuven, Belgium, in September 2016 to critically evaluate all aspects of colonic motility from bench to bedside. The conference had three objectives: appraise the current knowledge of normal colonic motor patterns (CMPs) in humans and in animal models gathered both in vitro and in vivo; critically evaluate commonalities and differences between animal models and humans; and generate agreement on terminology and definitions that can be applied to the study of colonic motility among basic scientists and clinicians, as well as among adult and paediatric gastroenterologists (Box 1).

This Consensus Statement provides a conceptual and methodological framework to expand studies on colonic motility, both in experimental animals and humans. This work is intended not only to facilitate the development of new drugs to treat common colonic motility disorders, such as chronic constipation and irritable bowel syndrome (IBS), but also to pave the way for the development of appropriate diagnostic and therapeutic algorithms for management of paediatric and adult patients.

Box 1 Fundamental outstanding questions The consensus meeting raised a number of fundamental questions about our current knowledge of the colon and colon motility that are unanswered but are relevant to the successful integration of basic science and clinical science.

**1. What is the role of central inputs in colonic movements?**

Propulsion of colon contents and subsequent emptying (defecation) involve different levels of input from the central nervous system depending on diets. Omnivores, such as rats and humans, defecate at longer intervals than herbivores, such as rabbits and guinea pigs, and have greater dependence on neural inputs from the central nervous system and on environmental and social influences. Hence, the role of extrinsic innervation in humans in the generation of normal motor patterns and pathophysiology needs further study.

**2. What is the nature of haustra?**

The functional and anatomical nature of haustra in both the animal colon and the human colon remains unclear. Chen et al.^21^ concluded that “the two definitions come down to seeing haustra as passively formed by contracted taenia or actively formed by circumferential muscle contractions”, which leads to the question whether haustral boundaries in humans are fixed or are caused by functional slowly advancing circular muscle contractions. The persistence of folding at particular sites of the colon after death would favour the first hypothesis, whereas the disappearance of the haustrations during mass movements and their documentation as migrating motor complexes in the proximal colon of herbivores would favour the second hypothesis.

**3. What are the characteristics of colonic motility under physiological conditions?**

Much of our current knowledge on colonic motility in humans has been acquired after a bowel preparation and anaesthesia or local sedation that are necessary for the placement of manometric catheters. Thus, the challenge of establishing the characteristics of the motor patterns and their role in transit and absorption under more-physiological conditions remains.

## Methods

The decision to initiate a consensus effort to bring together basic scientists and adult and paediatric gastroenterologists and conduct the first ‘translational’ consensus in this area was taken by a small core group (M. Corsetti, M. Costa and J. Tack) that prepared and initiated the process, after a meeting in Rome in September 2015. Because no precedent for such efforts existed and the topic was not readily amenable to traditional scientific investigation, an approach based on expert-based consensus was chosen. The core group identified a group of eligible contributors who have considerably contributed to the current knowledge in this field over several years and who were selected and invited to participate in the process. The selection process took three characteristics into account: diversity in research expertise (study of colonic motility in animal models, in human samples in vitro, in adults and children with manometric but also nonmanometric techniques); affiliations with different societies (United European Gastroenterology, American Gastroenterological Association, European and American societies); and diversity in region. All invited participants agreed to participate and were supportive of the scope and general aims of the project, leading to the consensus group comprising the current 15 members.

The core group identified five areas of interest, corresponding to the main sections of this Expert Consensus Statement, which were described in a working document. This document was circulated to each member and it was decided that each group would work on their assignments from October 2015 to prepare a review document to be presented and discussed in a face-to-face meeting in Leuven in September 2016. Five leaders were identified to coordinate the work of each group and to facilitate the interaction with the core group that coordinated the consensus. During this period, the members of each group interacted with each other via e-mail to prepare their document that was then circulated to members of the other groups 2 months before the consensus meeting.

During the face-to-face meeting, all five documents were briefly presented, extensively discussed, and revised. Subsequently, the members of the consensus group drafted five separate text segments, which were merged into a first draft of the manuscript which was then circulated to all participants in March 2017. All members commented on the content and wording, and areas of disagreement were identified, discussed and adapted if required. This process resulted in optimization of the language, harmonization of terminology and restructuring of the manuscript. An updated version of the manuscript was generated and circulated for evaluation in July 2017. All panel members endorsed this version, which was finalized into the document submitted for publication.

M. Corsetti had the idea of the consensus. M. Corsetti, M. Costa and J. Tack organized the consensus, collated the different parts and provided critical revision of the manuscript. M. Costa, J. D. Huizinga and R. Lentle performed the review and wrote the section concerning the colonic motility as studied in animals. N. J. Spencer and M. Jimenez performed the review and wrote the section concerning the colonic motility as studied in isolated human colon. M. Corsetti, P. Dinning, G. Bassotti, J. D. Huizinga and S. Rao performed the review and wrote the section concerning the colonic motility as studied in adult humans by means of colonic manometry. C. Di Lorenzo, M. Benninga and O. Borrelli performed the review and wrote the section concerning the colonic motility as studied in children by means of colonic manometry. A. E. Bharucha, P. Dinning and R. Spiller performed the review and wrote the section concerning the colonic motility as studied in humans in vivo by means of non-manometric techniques. J. Pannemans and A. Thys collected the minutes of the consensus meetings, prepared the figures and the references of the manuscript and helped M. Corsetti, M. Costa and J. Tack.

## Considerations for techniques

Suitable recording methods must be applied to establish which processes underlie the generation of different motor patterns. Since the development of advanced electrophysiological techniques, spatiotemporal mapping techniques and HRM, a considerable body of evidence has accumulated on the neurogenic (dependent on neuronal pathways) and myogenic (dependent on smooth muscle activity) components of CMPs. Most CMPs are the result of the interaction between neurogenic and myogenic processes, as both neurogenic and myogenic mechanisms operate concurrently. Depending on the main process underlying the motor patterns, the terms neurogenic, myogenic or neuro-myogenic are used in this article.

The methods that are most frequently used and suitable to describe motor patterns include mechanical recording with isometric or isotonic transducers, strain gauges, intraluminal pressure sensors, and some correlations with electrical extracellular or intracellular recordings. Methods of recording movements with video, fluoroscopy or cineradiography, and different forms of analysis of changes in shape of the colonic wall have been introduced over the past two decades^10–13^.

Motor patterns can obviously be better described using more intact segments of the colon and multiple recording sites (for example, with spatiotemporal maps) than when using small tissue strip preparations and recording at one location along the colon. When studying CMPs, distinguishing between simply observing motor activities for some period of time (naturalistic observations) and stimulus-induced motor patterns (experimental physiology) is important. An example of naturalistic motor activity is the occurrence of high-amplitude propagating contractions (HAPCs) recorded during colonic manometry. An example of an experimental physiological motor pattern are the HAPCs evoked by intraluminal bisacodyl^14^. These two different approaches to investigation are equally valid for examining colonic motility, and provide complementary information.

The nomenclature of specific colonic motor events has been widely discussed. Frequently used terms include contractions, sequences or pressure waves. The consensus panel agreed that any of these terms are acceptable and authors are encouraged to indicate the equivalent terms explicitly.

CMPs can be highly modulated by, or even be dependent on, extrinsic neural connections via visceral sensory pathways and efferent sympathetic and parasympathetic pathways. To what extent motor patterns are generated by neurogenic and myogenic mechanisms, even in the absence of extrinsic inputs, is not yet clear. In animals, a far higher number of studies have been performed on isolated preparations (in vitro and ex vivo are used as equivalent terms) than in live animals (in vivo). By contrast, in humans, far more observations have been made in conscious individuals than in isolated preparations of human colon. Some evidence indicates that many motor patterns described in vivo, such as neurogenic peristaltic contractions and slow-wave-generated myogenic phasic contractions, can be observed in isolated preparations under suitable conditions^15,16^. Hence, the consensus group widely agreed that CMPs could, in principle, be observed both in vivo and in vitro.

## Colonic motor patterns in animals

This section summarizes the different motor patterns described in the literature. We propose common terminology for CMPs and identify areas for further research into underlying mechanisms.

In the colon of omnivores (mouse, rat, dog, pig or human) the anatomical distinction between the proximal and distal colon is less marked than in herbivores^17^, although a separate caecum is present. The human colon is surprisingly more complex than that of other omnivores. It has a relatively small caecum and three taeniae, which extend from the proximal colon all the way to the sigmoid colon, where they fuse to form a single longitudinal muscle layer that continues into the rectum^17^.

The colons of the rat and mouse do not have separate taeniae, and functional differences along their length are still being investigated. The most detailed analysis of colonic movements and comparisons of differences in its anatomical components have been performed in two herbivores: the rabbit and the guinea pig^18–21^. The interest in these two species, particularly in the rabbit, stems from the similarity of its triple taeniated proximal colon to that of the major part of the human colon. In these two herbivores, the anatomy of the large intestine shows marked anatomical variation along its length, with a separate caecum. The shaping of the particulate content of the colonic digesta commences in the proximal colon. By the time it reaches the colonic flexure, the solid residue is moulded into recognizable soft faecal pellets (scibalae). Distal to this transition area, and readily recognizable in herbivores such as rabbits and guinea pigs, pellets are found along the entire distal colon and rectum^18,22–24^. Although no obvious anatomical correlate to the flexure in herbivores exists in the human colon, the structure of the ascending, transverse and descending human colon seems to correspond to the proximal colon in herbivores. The functional equivalent of the flexure would then correspond to the sigmoid-rectal junction. Compelling evidence for this comparison requires functional confirmation.

### Myogenic colonic motor patterns

**Fig. 1:**
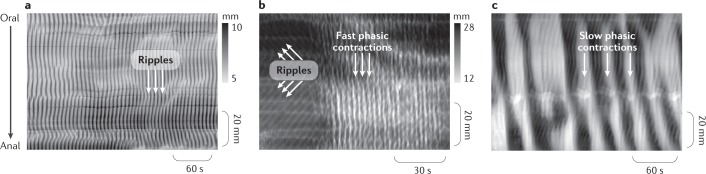
Myogenic motor patterns in animals. The images show graphical representations of wall motion captured by video recordings of a colon segment in an organ bath. Each frame of the video is converted to a greyscale image that maps changes in colon diameter. In these diameter maps (DMaps), darker shading represents an increased diameter (dilation) and lighter regions represent a reduced diameter (contraction). The three DMaps show the three main myogenic (non-neurogenic) patterns of motor activity in the colon of different animal species (in vitro isolated preparations). **a** | In the colon of most experimental animals, chaotic shallow contractions, termed ripples, are generated by a network of pacemaker cells at the submucous border (interstitial cells of Cajal (ICC) submucosal) acting on the circular muscle to elicit slow waves. In this example from a rabbit distal colon, these ripples become prominent once the neural activity is blocked by tetrodotoxin^20^. **b** | In some species, faster ripples have been recorded, which seem to be generated by a net of pacemaker cells located at the myenteric plexus level (ICC myenteric). In this example from the rabbit proximal colon, fast phasic contractions appear following application of hexamethonium, which blocks nicotinic receptors^24^. **c** | Slow phasic contractions have been recorded, for example in the rat colon after blocking neural activity with lidocaine and then applying the cholinergic agonist carbachol; whether these slow myogenic contractions exist in all species remains to be determined^37^. Part **a** adapted with permission from ref.^20^, The American Physiological Society. Part **b** adapted from ref.^24^, Springer Nature Limited. Part **c** adapted from ref.^37^, CC-BY-4.0 https://creativecommons.org/licenses/by/4.0/.

All the species studied seem to have at least three pacemaker cell systems in their colon. Electrical oscillations in smooth muscle are present throughout the digestive tract and are driven by pacemaker cells, now unequivocally identified as the interstitial cells of Cajal (ICC)^25–27^. In the colon, the network of ICC located near the submucosal border (the ICC of the submucosal plexus (ICC-SMP) and the ICC of the submucosa (ICC-SM)) generates electrical oscillations in the circular muscle, also called slow waves. Their frequency varies depending on the species, with increasing frequency in smaller species. For example, slow waves occur at 2–6 cycles per minute (cpm) in the human colon, but at 15–18 cpm in the mouse colon. These electrical oscillations propagate into the musculature and generate rhythmic contractions when the smooth muscle action potential threshold is reached (Fig. 1). The motor pattern generated is referred to as ripples.

D’Antona et al.^19^ defined ripples in the proximal colon in the guinea pig as orally and aborally propagating shallow circumferential contractions of the circular muscle, which are insensitive to tetrodotoxin. Lentle et al.^24^ and Dinning et al.^20^ also described them in the rabbit proximal and distal colon as contractions with varying levels of spatial coordination. All investigators who recorded colonic movements either in the conscious rabbit^23^ or in isolated rabbit colon preparations^8,20,21,24^ described ripples within the bulging haustrations, moving in either direction, although sometimes they were described as being preferentially propagated orally^23,24^. Ripples in the colon of herbivores seem to be involved in mixing and perhaps in retarding fluid progression, but not in bulk propulsion of contents.

The complex and chaotic nature of ripples probably results from the spontaneous fluctuation in membrane potential generated by contiguous nets of ICC pacemakers that generate different frequencies of oscillations within populations located at different sites^28^. The conceptual frame most suitable to describe the nature of this myogenic motor pattern is an active medium consisting of loosely coupled individually oscillating pacemaker cells^29^. As expected from this conceptual model, these oscillations propagate at varying distances along the colon both aborally and orally and annihilate each other when they collide^19,21,30^.

In the colon, the network of ICC at the boundary between the circular and longitudinal muscle (variously termed ICC myenteric plexus (ICC-MP), ICC myenteric (ICC-MY) or ICC Auerbach plexus (ICC-AP)) generates higher frequency oscillations than the ICC-SMP and ICC-SM that drive both muscle layers^31^. These oscillations were originally described as myenteric potential oscillations in dogs and humans^31,32^. These high-frequency electrical oscillations of smooth muscle membranes generate rhythmic contractions and are referred to as high-frequency ripples when they activate neighbouring muscle layers to their contractile threshold. High-frequency ripples, also termed fast propagating contractions in some publications, have been described in intact segments of the colon in rats and rabbits^21,33^. They are not abolished by drugs that block neural activity (for example, tetrodotoxin)^24,33^. These high-frequency ripples appear synchronous over some distances and occur at higher frequency than the slow waves in the same species^21,24,33^. They consist of synchronous longitudinal and circular contractions^24^ that do not have a ring-like appearance^21^. They traverse patterns of ripples without annihilating them^24^, suggesting that they are driven by a pacing network distinct from the one that generates ripples that are not high frequency. Summation (amplitude–amplitude interaction) or phase amplitude cross-frequency coupling between fast oscillations of the ICC-MP and slow waves from ICC-SMP can occur^31,34^. Lentle et al.^24^ suggested that the frequency of high-frequency ripples seemed to vary spontaneously between several discrete values.

The mechanical role of these high-frequency ripples in normal movements is uncertain. By themselves, they are unlikely to be mechanically effective in mixing or propulsion. However, the high-frequency ripples often occur in clusters and might become propulsive^6^. Furthermore, when slow-wave-driven ripples occur simultaneously with clusters of high-frequency ripples, the intraluminal pressure generated becomes greater than if either ripple occurs alone^20,35^.

Slow phasic contractions are a third type of much slower myogenic contractile activity that has been described in some species. Slow phasic contractions are caused by cyclic smooth muscle depolarizations that can be recorded both in the longitudinal and circular muscle^28,36^. Slow phasic depolarizations and contractions are recorded in tissue devoid of ICC-SMP, and consequently this myogenic activity probably originates from within ICC-MP. Such myogenic contractions have been well demonstrated in the rat colon^28,33,37^ and rabbit colon^20,21,24,33^. In guinea pigs, similar slow myogenic contractions occur at the colonic flexure^38^. However, slow myogenic contractions were not detected in full segments of the distal colon of the guinea pig even after direct stimulation of the muscle^33^. Possibly, these myogenic contractions are dependent on specific conditions.

The slow phasic contractions are more susceptible to modification by enteric neural inputs than the slow waves^39^. Inhibitory neurons inhibit slow phasic contractions whereas excitatory neurons enhance them, and slow phasic contractions can possibly be recorded inside motor complexes^39^. The possibility that interacting pacemaker cells could also generate such slow-frequency contractions has been suggested^40^. Distension is probably still required to generate these myogenic contractions after neural blockade, and in vivo cooperation between these myogenic processes and enteric neural circuits to generate complex motor patterns is likely^37^.

### Neurogenic colonic motor patterns

**Fig. 2:**
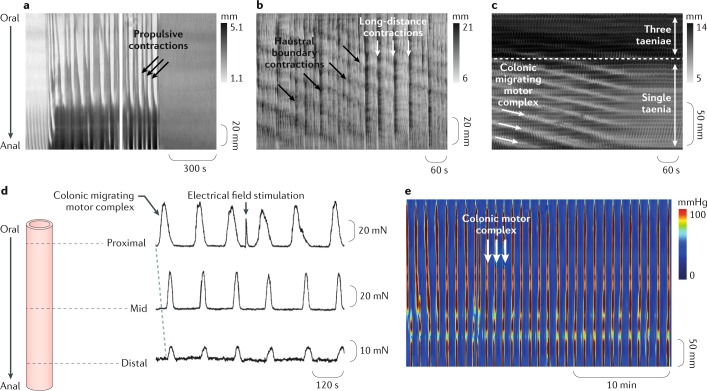
Neurogenic motor patterns in animals. Images **a**–**c** show examples of neurogenic motor patterns expressed as spatiotemporal maps showing changes in diameter (DMaps) of the excised colon of different animal species in vitro. Two main neurogenic motor patterns have been recognized. The first is neural peristalsis (consensus term) triggered by distension, which was described as propulsive contractions in the guinea pig distal colon^33^ (part **a**) and subsequently as long-distance contractions in the rabbit colon^21^ (part **b**). The second motor pattern present in the rabbit proximal colon consists of very slowly propagating contractions, representing the neural colonic motor complex^20^ (part **c**) and subsequently as haustral boundary contractions or progression (part **b**). In the complete mouse colon^199^ (part **d**) and in the guinea pig distal colon^81^ (part **e**) colonic motor complexes occur in distended segments. Neurogenic peristalsis is triggered and sustained by content, whereas the colonic motor complexes are generated as regular motor activity slowly traversing long segments of colon or appearing even in the absence of any propulsion of contents. Part **a** adapted with permission from ref.^33^, The American Physiological Society. Part **b** adapted with permission from ref.^21^, The American Physiological Society. Part **c** adapted with permission from ref.^20^, The American Physiological Society. Part **d** adapted with permission from ref.^199^, Wiley-VCH. Part **e** adapted with permission from ref.^81^, Wiley-VCH.

In all animal species, both in vivo and in vitro, strong evidence exists that distension of the colon to a suitable extent (regardless of content consistency) initiates neurogenic propulsive movements that propel the contents aborally (Fig. 2).

In their classic preparation of the colon of dogs, maintained under deep irreversible anaesthesia, Bayliss and Starling^41^ used a solid bolus and described propulsive movements that were evoked as peristaltic contractions or peristaltic waves. Several investigators have since shown colonic propulsion of boluses in isolated preparations in mice^42,43^, in guinea pigs^44,45^ and in rabbits^46,47^, as well as in conscious dogs^48–53^. Similar propulsive contractions have also been described in vivo in cats^54^, in monkeys^55^, in ferrets^56^ and in rats and mice^57,58^. Several of the investigators named such propulsive movements giant contractions.

Distension of the proximal colon triggers large propagating contractions that propel content aborally. These movements have also been termed mass movements or mass peristalsis^24^ and are similar in nature to those observed in the rabbit colon in vivo by Ehrlein and colleagues^23^. Similar propulsive motor patterns occur in the proximal and distal colon of guinea pigs when it is markedly distended by liquid. In these latter studies, the activity was called neural peristalsis or antegrade neurally dependent peristaltic contractions^18,19^. In the isolated cat colon, such propulsive movements were described as net aboral propulsion^59^. In the isolated colon of the guinea pig, rat and mouse, fluid distension triggers rapid fluid propulsion variously termed neural peristalsis, or long-distance contractions^33,42,60,61^. A general consensus exists that these propulsive movements, initiated by a bolus or general distention, are generated by enteric neural circuits and can therefore be regarded as neural in origin (neurogenic) and are described as peristalsis or the peristaltic reflex^45,62–67^. The term peristaltic reflex has often been used to refer to the activation of polarised enteric reflexes^45,68^, whereas peristalsis characterizes a sequential activation of the polarised reflexes and should therefore be regarded as an ongoing behaviour^69,70^. Thus, a suitable consensus modern term to describe these movements is neural peristalsis. The amplitude and propagation speed of the contractions underlying these propulsive movements varies depending on the contents (solid, viscous or liquid), as discussed later.

In 1899, Bayliss and Starling^71^ proposed that the propulsion of a bolus is a result of the activation of polarized enteric neural pathways leading to oral contraction and anal relaxation. The displaced content was thought to be responsible for the subsequent activation of neural pathways further down the intestine ahead of the advancing bolus. This original concept of the peristaltic reflex has been modified to incorporate actual evidence of polarity of neural activity into what is now proposed as the neuromechanical loop hypothesis^72^. This advance in understanding was made possible through simultaneous recording of pressure and diameter changes. This enabled the calculation of the mechanical states of the intestinal muscle to be established, distinguishing active (due to active contraction or relaxation) from passive changes^10^. Two components of the neuromechanical loop are distinguished: a bolus-evoked graded activation of polarized enteric pathways (the neural part) and the oral contraction and anal relaxation (the mechanical part)^10,33^. Both neural and mechanical components are adaptable, and this functional loop leads to variable aboral propulsion of the bolus. Distension of a new portion of the gut then initiates a new loop process. The validity of this model was demonstrated when it was shown that the speed at which the bolus moves was dependent on its size, shape and viscosity^18^. Thus, colonic propulsion is not simply due to an invariant, stereotyped peristaltic reflex; CMPs adapt to the physical properties of the contents.

Some descriptions of neural retrograde peristaltic contractions (antiperistalsis; neural retrograde propagating contraction) also exist. Antiperistalsis described by Bayliss and Starling^41^ in the rabbit colon was demonstrated by Dinning et al.^20^ to be neurogenic but insensitive to blockade of nicotinic transmission. This motor pattern is potentially important, but its function remains largely unclear and will require further investigation.

### Neurogenic colonic motor complexes

Repetitive events of motor activity have been widely reported in the colon of most species (Fig. 2). In 1926, Welch and Plant^73^ showed in the canine colon that periods of contractile activity existed, lasting 5–15 min and recurring at regular intervals of 15–30 min. Numerous other studies in dogs^51,74–76^, most notably those by Sarna et al.^50,77^, and other species, including in rats^78,79^, in rabbits and in larger mammals, such as horses^80^, have shown similar findings.

Clear examples of repetitive motor activity at a level similar to that observed in vivo have been recorded in isolated preparations of rabbit proximal colon that was distended by liquid (Krebs solution)^23^. Overall, repetitive propagating contractions slowly moving anally along the colon have been well described under liquid distension by three different laboratories, albeit with different terminologies (Lentle et al.^24^: haustral progression; Dinning et al.^20^: colonic migrating motor complex; Chen et al.^21^: haustral boundary contractions). All investigators reported that blocking neural activity abolished this motor pattern. This neurogenic activity interacts with the myogenic ripples to give the feature of advancing clusters of rhythmic contractions (motor complexes). Because of this interaction between neurogenic and myogenic mechanisms, the motor patterns can be regarded as neuromyogenic. In guinea pigs, similar slowly migrating motor complexes have been observed in the proximal colon and seem to be involved in the early formation of soft pellets^81^. Based on these findings, the main function of these motor complexes, slowly migrating aborally in herbivores, seems to be initial sequestration and aboral progression of the soft contents of the proximal colon and their transformation into more solid (viscoelastic) faecal pellets. Similar motor patterns probably also occur in the colon of other species.

In most species, fixed distension of the colon with a tethered pellet or localised stretch with an isometric transducer will generate similar neurogenic repetitive motor events. The best known examples of such motor activity have been originally described as colonic migrating motor complexes (CMMCs) that occur in a full-length isolated mouse colon^82^ and in the guinea pig colon^45,81,83^. However, under these conditions of maintained distension, motor complexes can propagate in both retrograde and anterograde directions. In 2017, the electrical activity in the colonic smooth muscle that underlies CMMCs was recorded from isolated full-length colon preparations. These cyclical patterns of myoelectric activity in the mouse colon have been referred to as neurogenic spike bursts, which occur at the same frequency as CMMCs^84^. To preserve neurogenic spike bursts and CMMCs in vitro, considerable lengths of colon are required. Full neurogenic spike bursts were only recorded in 30-mm-long preparations and were absent in 10-mm preparations^84^.

In many instances, it is impossible to resolve whether individual motor complexes actually propagate or simultaneously occur over a region of the bowel. For example, a guinea pig colon distended by a manometric catheter showed repetitive neurogenic increases in intraluminal pressure that propagated in either direction or occupied up to 25 cm of colon simultaneously^81^.

As a less committed term that is still descriptive, we suggest using the generic term colonic motor complexes to describe any neurogenic repetitive contractions in experimental animals, whether they migrate or not. The term colonic migrating motor complexes should then be restricted to complexes that are proven to migrate.

## CMPs in the isolated human colon

The human colon is one of the least understood organs of the body, largely because of difficulty with access, both in vivo and in terms of availability of human resection specimens for experimental studies. Most studies on isolated human colon have used very small segments of colon, typically from 1 to 2 mm to a few centimetres in length^32^. These small segments are useful for studying the myogenic activity in the colon but are less useful for understanding the neurogenic motor patterns, as they are of an insufficient length to determine the characteristics of propagation.

### CMPs in isolated colon strips

**Fig. 3:**
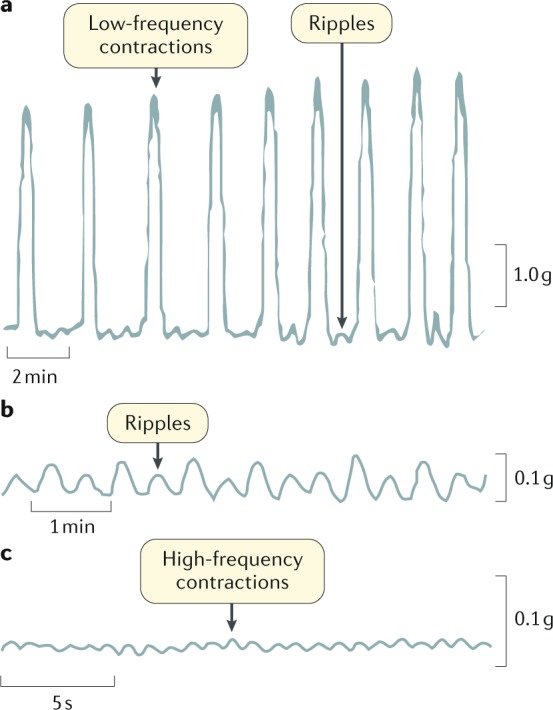
Motor patterns in isolated strips of the human colon. **a** | Low-frequency contractions superimposed with intermediate-frequency motor events or ripples. **b** | Intermediate-frequency motor events or ripples. **c** | High-frequency contractions. Different time and amplitude scales were used to optimally visualise each type of contractile activity.

Most of the studies on small strips of human colon have reported spontaneous contractions that occur at three different frequencies: high-frequency contractions (frequency usually >10 cpm) of low amplitude probably due to ICC-MP; intermediate frequency contractions (~2–4 cpm) probably due to slow waves; and low-frequency high-amplitude contractions (<1 cpm) that persist in samples devoid of ICC-SMP, possibly generated as a result of stretch in ICC-MP and potentiated by other excitatory inputs^39^ (Fig. 3; Table 1). These three activities are considered the respective correlates of high-frequency ripples, ripples and slow phasic contractions recorded in the isolated intact colon from the animal studies discussed earlier.

**Table 1:** Motor patterns in isolated strips of human colon

Name of the colonic pattern	Rae et al. (1998)^32^	Carbone et al. (2013)^85^	Mane et al. (2015)^200^	Possible origin	Correlation with animal studies
High-frequency contractions (low amplitude)	15–18 cpm	20 cpm	Not identified	• ICC-MP • Myenteric potential oscillations	High-frequency ripples
Intermediate-frequency contractions (low amplitude)	1.6–3 cpm	3.8 cpm	2–4 cpm	• ICC-SMP • Slow waves	Ripples
Low-frequency contractions (high amplitude)	0.5–0.9 cpm	1 cpm	0.3–0.6 cpm	• ICC-MP • Cyclic depolarizations	Slow phasic contractions

cpm, cycles per minute; ICC, interstitial cells of Cajal; ICC-MP, ICC myenteric plexus; ICC-SMP, ICC submucosal plexus.

### CMPs in isolated flat colon preparations

Recordings of circular muscle contraction in isolated preparations of human colon that were opened lengthwise and mounted flat showed slow spontaneous contractions, termed slow phasic contractions^85^, occurring at intervals of 0.5–5 min. Electrical activation of enteric neural pathways could initiate these contractions prematurely, resetting their rhythmicity^85,86^. Similar slow phasic contractions persisted following blockade of all neural activity with tetrodotoxin^86^.

### CMPs in the isolated colon

**Fig. 4:**
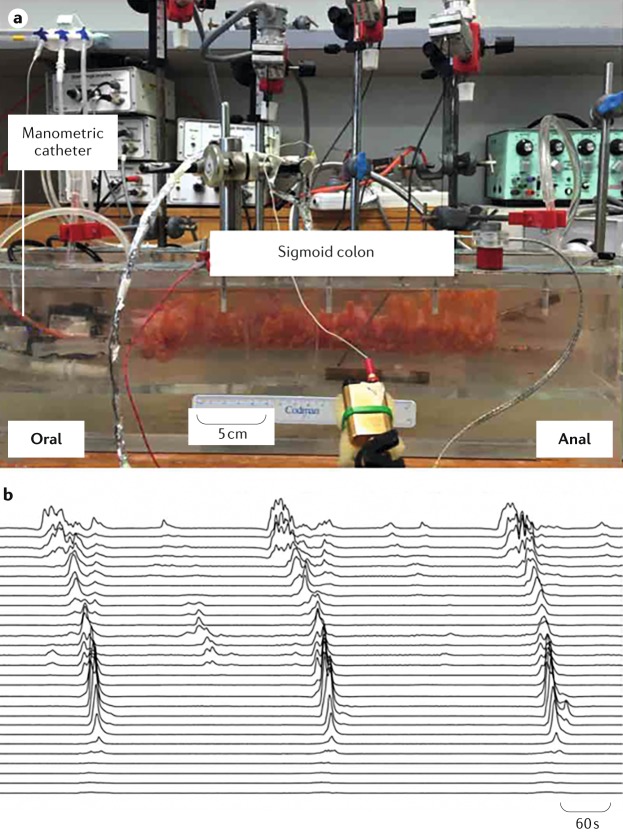
Colonic motor patterns of an excised section of human sigmoid colon. **a** | The excised sigmoid colon is placed into an organ bath filled with an oxygenated Krebs solution maintained at 37°C. Motor patterns are recorded by a high-resolution manometric catheter attached to a rod at the base of the preparation. **b** | A series of propagating pressure waves recorded from the section of the colon. Three large propagating contractions start at the oral end and move towards the anal end of the segment.

Two studies have recorded activity from the isolated human colon in vitro^86,87^. These studies show two major types of motor activity: repetitive sequences and single propagating sequences (Fig. 4). To establish a harmonized terminology, we suggest using the terms repetitive motor patterns and single motor pattern (Table 2). Both sequences can be initiated at any site along the colon and propagate over considerable distances in either anterograde, retrograde or both anterograde and retrograde directions. They can readily be evoked by enteric nerve stimuli or luminal distension^86^. They are considered the correlates of the previously described colonic motor complexes recorded in animal studies discussed earlier.

**Table 2:** Motor patterns in isolated human colon^86^

Name of the colonic pattern	Interval between events	Directionality of propagation	Extent of propagation	Site of initiation	Trigger	Stimulus
Repetitive motor pattern	4.0 ± 0.6 min (0.25 cpm)	Anterograde, retrograde or simultaneous	Variable; usually >20 cm	Variable; at different sites along the length of the colon	Spontaneous	Enteric nerve stimulation
Single motor pattern	Single event	Anterograde or retrograde	Variable; usually >20 cm	Variable; at different sites along the length of the colon	Spontaneous	Enteric nerve stimulation and balloon distension

cpm, cycles per minute.

## CMPs in adults by manometry

Previous studies, using manometric catheters with recording sites at ≥7.5-cm spacings, have demonstrated that colonic motor activity in healthy individuals is represented mainly by nonpropagating activity, interspersed with propagating contractions of low amplitude and of high amplitude occurring in limited numbers of 2.4 ± 0.1 and 0.4 ± 0.1 events per hour, respectively^88,89^. Both nonpropagating and propagating activity is reduced in patients with chronic idiopathic constipation^90,91^. However, as no qualitative or quantitative data exist that unequivocally differentiate normal from abnormal colonic function^5,92^, the role of colonic manometry in the management of these patients remains unclear^5^.

Surprisingly limited knowledge exists regarding the anatomical or functional nature of the haustra in the human colon. It has previously been argued that they are fixed anatomical features, or that they are transient contractions of the intertaeniae circular muscle, resulting in the typical triangular narrowing observed in colonoscopies^93^. However, as the haustrations disappear during mass movements^94,95^, they are probably functional contractions rather than fixed anatomical features.

The introduction of HRM, in which the distance between the intraluminal pressure sensors has been decreased to one sensor every 1–2.5 cm, has been found to increase the accuracy of detection of CMPs, in particular that of low-amplitude propagating activity^96^. Currently, only a few studies have used HRM to monitor colonic motility in adult humans. Hence, the following section summarizes CMPs identified in healthy individuals both by conventional manometry and by HRM (Table 3).

**Table 3:** Colonic motor patterns in healthy humans^6,7,109,111^

Name of the colonic pattern	Amplitude	Frequency	Response to waking	Response to meal	Stimulant	Suppressant
Simultaneous pressure increases	Low	≤1–2 cpm if repetitive	Increase	Increase (meal of ~400 kcal)	Neostigmine (i.v.), prucalopride (p.o.)	Not known
Haustral boundary pressure transients	Low	3 cpm	Not known	Not known	Not known	Not known
Intrahaustral activity	Low	3 cpm	Not known	Increase	Not known	Not known
Cyclic propagating motor pattern	Low; mainly retrograde	2–6 cpm	Not known	Increase (meal of ~700 kcal)	Not known	Not known
Short single motor pattern	Low	Not repetitive	Increase	Increase	Not known	Not known
Long single motor pattern	Low	1 cpm if repetitive	Not known	Not influenced	Polyethylene glycol (p.o.) and linaclotide (p.o.)	Not known
Slow retrograde motor pattern	Low	Not repetitive	Not known	Not influenced	Not known	Not known
High-amplitude propagating contractions	>75 mmHg	Can be repetitive	Increase	Increase (~1–2 h after meal)	Yohimbine (i.v.), glycerol (i.c.), oleic acid (i.c.) and bisacodyl (i.c. and p.o.)	Lidocaine (i.c.) and phloroglucinol (i.v.)

cpm, cycles per minute; i.c., intracolonic; i.v., intravenous; p.o., per os.

### Simultaneous pressure increases

**Fig. 5:**
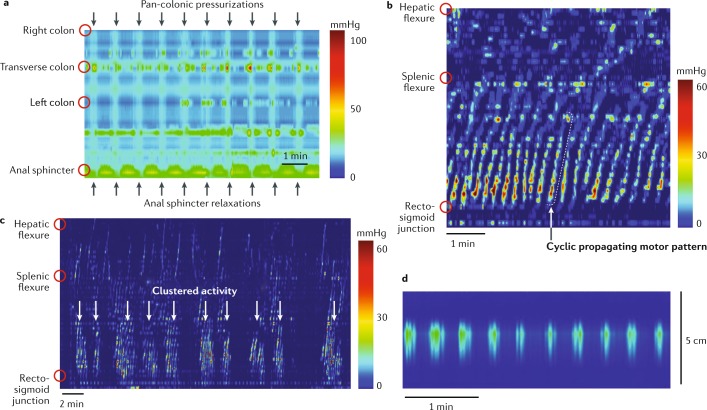
Colonic motor patterns frequently identified by HRM in adults. The introduction of high-resolution manometry (HRM) increased the accuracy of the detection of colonic motor patterns. The most frequently detected patterns in adults are simultaneous pressure increases, cyclic propagating motor patterns and haustral activity. **a** | Examples of simultaneous pressure increases (pan-colonic pressurizations)^7^. These are characterized by simultaneous pressure increases recorded across all recording sensors and are associated with relaxation of the anal sphincter. **b** | The cyclic propagating motor pattern, shown as a spatiotemporal colour plot, recorded in a healthy adult colon. This activity increases after a meal, originates at the rectosigmoid junction and propagates primarily in a retrograde direction (anal to oral). **c** | Cyclic propagating motor patterns can also occur in clusters spaced at 1–4-min intervals. These clusters appear after the consumption of a high-calorie meal. **d** | Intrahaustral activity often has a frequency of ~3 cpm in ≤5 consecutive sensors and, therefore, seems to be activity within a haustrum^8^. Part **d** adapted from ref.^8^, CC-BY-4.0 https://creativecommons.org/licenses/by/4.0/.

These CMPs were identified using conventional manometry but were tentatively considered to be artefacts caused by contractions in the wall of the abdomen^97–101^. However, the same simultaneous pressure increases were recorded in studies that used HRM to concurrently evaluate colonic and anal sphincteric activity^7^ (Fig. 5). With HRM, the pressure increases were recorded across all manometry channels and were temporally associated with relaxation of the anal sphincter. Importantly, no accompanying changes in abdominal wall electromyography activity were recorded^6–8^. By contrast, colonic lumen pressure increases that were associated with contractions in the abdominal wall or other artefacts were associated with pressure increases in the anal sphincter and with electromyographic activity in the abdominal wall^7^. The simultaneous pressure increases associated with anal sphincter relaxations were termed colonic pressurizations. In some colonic manometry studies in healthy adults, these colonic pressurizations were the most commonly recorded motor pattern and were associated with a desire to evacuate gas and/or gas expulsion^7,8^. These simultaneous pressure increases might be equivalent to the colonic motor complexes observed in the colon of mice and guinea pigs during maintained distension.

### Haustral boundary pressure transients

This motor pattern consists of sequences of highly rhythmic (3 cpm) transient pressure increases, which are recorded by single sensors but repeated at around every five sensors (hence, around every 5 cm) moving across these sensors^8^. They seem to represent haustral boundary contractions, corresponding to CMMCs in mice, in rabbits (also called haustral boundary progression^24^ or haustral boundary contractions^21^) and in guinea pigs (also called proximal colon migrating motor complex^18^). However, as limited knowledge exists regarding the haustra in the human colon, further work is needed to determine whether this activity corresponds to actual movements of haustrations.

The fact that rhythmic changes in intraluminal pressure in the human colon sometimes occur with a frequency of ~3 cpm suggests that they are due to the myogenic contractile activity termed ripples^8^. The rhythmic pattern commonly occupied ≤5 consecutive sensors and could, therefore, have been occurring within a single haustrum. The individual pressure wave sometimes propagated at 2 ± 1 cm/s in either an antegrade or a retrograde direction, consistent with its genesis in the propagation of slow waves^8^. Hence, these are likely to be intrahaustral myogenic contractions^8^ (intrahaustral activity), that is, ripples responsible for segmental activity as also described in the rabbit colon^21^.

### Cyclic propagating motor pattern

This CMP was the most commonly recorded propagating motor pattern in studies of the healthy adult colon using fibreoptic HRM^6^. The individual propagating contractions within this motor pattern had a frequency of 2–6 per minute, which suggests they result from ripples generated by myogenic slow waves amplified by neural excitatory inputs. Activity of a similar frequency has been recorded in the rectum using conventional manometry and was termed periodic rectal motor activity, as well as in the colon where it was termed segmental activity^88,98,102–104^. Each sequence of propagating contractions within the cyclic motor pattern travelled predominantly in a retrograde direction. Cyclic motor patterns were observed in the proximal and descending colon, but the majority (76%) were identified in the rectosigmoid colon.

### Low-amplitude single propagating contractions

These CMPs were identified in conventional manometric studies and are characterized as elevations in pressure <50 mmHg, propagating over at least three consecutive recording ports, usually in an aboral direction^88,100,101,105^. These motor patterns were recorded more commonly during the day and occurred at an approximate rate of 61 events per 24 h. They have been reported to be associated with the transport of luminal content, expulsion of intestinal gas and relaxation of the anal sphincter^89,106^. Subsequent colonic HRM studies characterized three subtypes.

The short single motor pattern is a propagating motor pattern that occurs in isolation, separated from other propagating motor patterns by intervals >1 min (refs^6,7^). This motor pattern could propagate in both anterograde and retrograde directions over short lengths of colon (~7 cm) at a velocity of 0.5 ± 0.3 cm/s. It originated primarily in the proximal colon (42%) or sigmoid colon (43%), and made up 24.2% of all propagating motor patterns.

Long single motor patterns comprise single pressure events that propagate over long distances^6,7^. These events are always separated by intervals >1 min when they occur repetitively. Most originate in the proximal colon (76%) and the remaining 24% originate in the proximal region of the descending colon. These contractions usually propagate in an anterograde direction. They can be distinguished from short single motor patterns since they propagate over considerably longer distances along the colon (40.8 ± 8.4 cm), often as far as the distal descending or sigmoid colon. Their incidence is increased by polyethylene glycol and linaclotide^107,108^. These events might be the correlates of the long-distance contractions or neural peristalsis observed in animal studies.

Slow retrograde motor patterns are rare but distinctive propagating motor patterns that are typically observed during fasting^6,7^. They travel at <0.5 cm/s and propagate over distances generally >40 cm, starting in the sigmoid colon and entering the transverse colon. They make up only 0.3% of all propagating motor patterns.

### High-amplitude propagating contractions

These CMPS are prominent and of diagnostic importance, but only occur between 4 and 23 times over the course of 24 h. This motor pattern consists of powerful propagating contractions that are associated with the mass movement of colonic content and with defecation^88,89,98,100,109–111^. Over the years, this motor pattern has been variously defined as pressure waves with amplitudes varying between 50 and >136 mmHg, lasting 10–30 s (refs^6,112^). This motor pattern extends at least 10–30 cm along the length of the colon and mostly originates in the proximal colon^6,112^. These events are probably the correlate of the neural peristalsis described in animal studies.

The stimuli that generate these contractions remain unclear. They might be triggered by distension of the lumen caused by intraluminal accumulation of colonic content. A postprandial increase in the number of HAPCs has been demonstrated in healthy adults in a number of studies. However, the number of postprandial HAPCs that are generated seems to depend on a number of factors. Studies performed within hours of a bowel preparation and catheter placement reported low numbers of postprandial HAPCs even in healthy subjects^113^. Once the colon begins to fill again by the following day, the postprandial count of HAPCs increases^114^. Ileal propagating activity has been reported to continue throughout the night^115^, and might augment colonic filling and activate the enteric circuits that generate HAPCs. HAPCs are suppressed during sleep, suggesting that their incidence is influenced by the central nervous system^116^.

Various pharmacological interventions influence the incidence of HAPCs in humans. Blocking the adrenergic alpha-2 receptor with yohimbine in a study applying colonic distension controlled by an electronic barostat increased the number of HAPCs^112^. Intraluminal application of glycerol, oleic acid or bisacodyl can also increase the incidence of this motor pattern^112^. Neither the administration of granisetron (a 5-hydroxytryptamine 3 receptor antagonist) intravenously or octreotide (a somatostatin analogue) subcutaneously affected the number or characteristics of HAPCs, whereas intraluminal lidocaine and intravenous phloroglucinol inhibited HAPCs induced by intraluminal glycerol^117^. Intravenous atropine did not inhibit spontaneous postprandial HAPCs^118^ and HAPCs were not elicited by powerful cholinergic stimulation induced by intravenous edrophonium chloride^119^.

## CMPs in children by manometry

Among the diagnostic tools available for the assessment of children with constipation that do not respond to medical and behavioural management, colonic manometry is currently advocated as the most informative modality for evaluating colonic neuromuscular function^3^. This test has been used in children to clarify colonic pathophysiology and direct clinical care for the past 25 years. Colonic manometry is also among the tests required for accomplishing level II training in paediatric neurogastroenterology and motility in the United States^4^. Minimum standards to be used in performing this test were suggested almost two decades ago^120^.

In children, colonic manometry is currently indicated to discriminate between colonic neuromuscular disorders and functional constipation to guide the surgical approach in those patients refractory to conventional medical treatment, to evaluate the colonic involvement in children with chronic intestinal pseudo-obstruction, to determine whether a diverted colon is suitable for reconnection and to clarify the underlying mechanisms responsible for the persistence of symptoms after surgery for either Hirschsprung’s disease or other anorectal abnormalities^120^.

Until 2013, most data about childhood colonic motility patterns had been accrued using water-perfused catheters with six to eight recording sites spaced 10–15 cm apart^121^. The analysis of the resulting motor events has focused traditionally on the presence or absence of a postprandial increase in overall motility and on the presence, absence and extent of propagation of HAPCs^120^. As healthy children cannot be studied for ethical reasons, the conclusion about what constitutes normal motility in children has been derived from studies in paediatric patients who in retrospect (based on their clinical characteristics and treatment outcome) are deemed to have functional constipation. Functional constipation in children is considered a consequence of a disordered behaviour in individuals who have developed stool withholding in response to the urge to defecate after experiencing an unpleasant defecation. The colon is thought to have a completely normal motor function in these children and the problem is usually resolved once the child realizes that it is more comfortable to release colonic content than to withhold it^122^. Some of these children, especially those refractory to behavioural and medical therapy, have received colonic manometry testing and the data from these patients have been reviewed and analysed to describe normal colonic motility in children of different ages^123^. This section reports how these established findings, which have been used in the past 25 years to study children in clinical practice, integrate with more recent descriptions of other motor events measured by HRM.

### High-amplitude propagating contractions

**Fig. 6:**
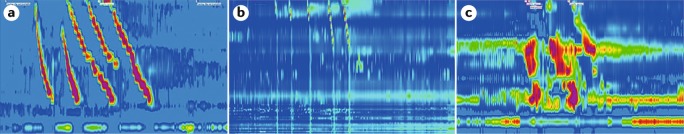
HAPCs in children. Normally and abnormally propagating high-amplitude propagating contractions (HAPCs) have been identified by high-resolution manometry in children. **a** | In normal HAPCs, the amplitude is >75 mmHg and the contractions propagate distally to the rectosigmoid junction. The anal sphincter relaxes concurrently to the HAPC. **b** | In abnormally propagating HAPCs, the contractions do not propagate beyond the transverse colon. **c** | An abnormal configuration of HAPCs with multipeaked waveforms and prolonged duration. This configuration has been associated with histological evidence of colonic neuropathy^121^.

The generally accepted definition for HAPCs in children is contractions with an amplitude of greater than 75 mmHg, a duration of greater than 10 s and propagation distance of 30 cm or more^92,124–126^ (Fig. 6). Normal HAPCs are expected not to propagate beyond the junction between the sigmoid colon and the rectum and are often associated with a relaxation of the anal sphincter (coloanal reflex)^127^. They typically occur following meals and upon waking, and can be induced by bisacodyl^128^. Octreotide and erythromycin do not induce HAPCs^129–131^.

HAPCs are generally classified based on their distance of propagation: fully propagating, when HAPCs reach the sigmoid colon; partially propagating, when HAPCs stop at the level of the splenic flexure or the descending colon; and absent, when no HAPCs are observed in the entire colon^125,132,133^. HAPCs can be further classified as normal or abnormal based on the morphology of pressure waves within the contraction sequences. Abnormal HAPCs have either two or more pressure peaks or a duration exceeding 30 s. Several aspects of HAPCs have been established based on paediatric studies^92,123–125,127,134–143^. They are reliably identified by different observers (with minimal interindividual variability)^134^ and are more common in younger children (their frequency is inversely correlated to age in a group of children aged 1–17 years)^123^. They are more common in patients who have had a distal colonic resection, such as after surgery for Hirschsprung’s disease, probably owing to the disruption of the inhibitory recto–colonic reflex^125,135^. HAPCs can be induced through infusion of saline and distention of the right colon^136,137^. Their presence suggests intact colonic neuromuscular function and studies based on the characteristics of HAPCs have been found to be predictive of medical and surgical outcome^132,137–139^. For example, patients with intact HAPCs are more likely to respond well to the administration of antegrade enemas or to the surgical resection of a dysmotile colonic segment. Abnormalities in HAPCs have been used to explain the pathophysiology of defecatory difficulties in various different medical conditions, such as imperforate anus, Hirschsprung’s disease, pseudo-obstruction and chronic constipation^92,124,140–143^. Bisacodyl-induced HAPCs are quantitatively and qualitatively similar to naturally occurring HAPCs^127^.

### Low-amplitude propagating sequences

Low-amplitude propagating sequences typically have an amplitude <50 mmHg and occur 40 to 120 times in a 24-h period in adults^144^. They occur considerably more often during the day than at night, and, similar to HAPCs, they increase in frequency following meals and upon waking. They have also been described in children and a considerable difference was found between patients with slow-transit constipation and healthy controls in the mean number of ascending, transverse and descending low-amplitude propagating sequences^121,145^.

### Patterns detected by HRM in children

Reports on using HRM to study colonic motility in children have only started to be published in the past 4 years. Several motility patterns other than HAPCs and low-amplitude propagating sequences in children presenting with constipation were described in 2016 (refs^121,146,147^). The characteristics of colonic motility in children with constipation were evaluated using HRM water-perfused catheters and were classified on the basis of definitions similar to those used in adults. The authors identified antegrade and retrograde cyclic propagating motor patterns, short single antegrade and retrograde motor patterns, and preprandial and postprandial long single motor patterns^147^. Similar to adults with constipation, children with constipation did not show the normal postprandial increase in the cyclic retrograde propagating motor pattern^147^. In addition, paediatric patients showed greater numbers of the antegrade propagating long single motor pattern during the preprandial phase^147^. The clinical significance of these findings is currently unclear. In one study, the presence of simultaneous pressure increases like those reported in adults was also observed in children with constipation^121^.

## Human colonic motility by other techniques

The major limitations of manometry are that pressure changes might not accurately represent colonic contractions or might not be associated with movement of intraluminal content. Hence, the techniques to assess colonic transit, tone and wall motion, which are relevant to colonic motor function, are evaluated here. Radiopaque markers, scintigraphy, a wireless pH and pressure recording capsule (SmartPill; Medtronics) and a telemetric capsule system (3D-Transit; Motilis Medica SA) have all been used to assess colonic transit. The SmartPill also measures pressure activity using a single sensor built into the device. The barostat balloon can measure colonic tone and phasic contractions. Over the past 4 years, MRI has been used to noninvasively assess unique parameters of colonic function that were previously inaccessible for measurement.

### Radiopaque markers

Whole-gut transit time and segmental colonic transit time can be estimated by counting the number of ingested markers remaining in the abdomen on abdominal X-rays. These techniques have been reviewed elsewhere^148^. The estimated whole-gut transit time also includes gastric and small bowel transit, which constitute a small proportion of the whole-gut transit time in healthy individuals and in patients with isolated slow-transit constipation, but constitute a larger proportion of the whole-gut transit time in patients who also have severely delayed gastric emptying (for example, in patients with diabetic gastroparesis). Hence, radiopaque markers can provide a surrogate measure of colonic transit. They remain the criterion standard, the cheapest and perhaps the most widely used technique to assess colonic transit in constipated patients.

Radiopaque markers, scintigraphy and the wireless motility capsule (SmartPill) provide comparable results regarding colonic transit. However, transit through the ascending and transverse colon was considerably shorter when measured by the radiomarker technique than by scintigraphy, probably because particle size influences regional colonic transit^149^. Beginning with the original reports of slow-transit constipation^150,151^, markers have been widely used to evaluate colonic transit in patients with constipation, and to a lesser extent in those with diarrhoea^152^. Markers have also been used to evaluate the correlation between colonic transit and stool weight^153^, and the effects of supplementing dietary fibre as well as physical exercise on colonic transit^154,155^.

### Scintigraphy

**Fig. 7:**
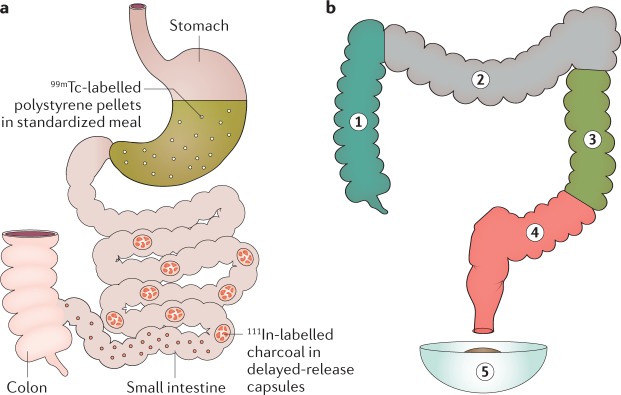
Scintigraphic assessment of gastrointestinal transit. **a** | Gastric emptying and small intestinal transit are assessed with ^99m^Tc-labelled polystyrene pellets, whereas colonic transit is measured with ^111^In-labelled charcoal in delayed-release capsules. **b** | Proportion of ^111^In counts in each of four colonic regions of interest and stool is multiplied by the appropriate weighting factor, ranging from 1 to 5.

Colonic transit scintigraphy is performed after patients swallow a capsule containing a radioisotope, typically indium (^111^In), which is tagged to charcoal pellets^156^. The capsule has a methacrylate coating that dissolves at a pH of 7.2–7.4, generally in the terminal ileum, releasing the isotope in the caecum^157^. Scintigraphy has been widely used to evaluate the effects of drugs and diseases on colonic transit. Measuring colonic transit with radiopaque markers takes 6 days compared with 48 h for scintigraphy. By integrating two isotopes, gastric, small intestinal and colonic transit can be simultaneously evaluated with scintigraphy^158–160^ (Fig. 7).

Early studies in humans that evaluated the relationship between colonic motor events and transit with simultaneous manometry and scintigraphy might have missed subtle movements because they used relatively slow scintigraphic acquisition rates, for example every minute^161^ or every 5 minutes^162,163^. Studies over the past 10 years have used faster image acquisition rates, for example every 10–15 s, with visual detection or quantitative assessment of colonic isotope movement^89,164^. These studies showed that movement of colonic content occurs in a stepwise manner over short distances, in both antegrade and retrograde directions, associated with propagating sequences^88,164^. In humans, 93% of propagated contractions were associated with propulsion of content^88,164^. Low-amplitude propagating sequences with amplitudes of 2–5 mmHg were as likely to be associated with movement of content as higher amplitude propagating sequences. However, of all the episodes of propulsion, fewer than half were associated with detectable propagating sequences. This finding might suggest that pressure waves are not essential for propulsion. The strength of this pressure–flow relationship seems to be region-dependent, being stronger in the transverse colon than in the caecum and ascending colon. In theory, the movement of colonic contents that is not accompanied by propagating colonic contractions might be explained by contractions at points remote from the recording sites, or motor events that do not substantially affect intraluminal pressure, such as longitudinal muscle shortening, nonlumen-occluding circular muscle contractions, or alterations in regional wall tone. Importantly, these combined manometry and scintigraphy studies used low-resolution manometry with recording sensors spaced at >7 cm intervals, which is much less accurate than HRM (sensors at 1 cm intervals) for identifying colonic propagating motor patterns^96^. Hence, the lack of association might also reflect, at least in part, technical constraints.

Retrograde sequences are generally associated with retrograde movements of colonic content. However, ~90% of retrograde flow episodes occur in the absence of retrograde sequences. About half of the retrograde flow events follow immediately after an antegrade movement, indicating frequent reflux of content back into the region from which it had just moved^164^. This subtle ‘to and fro’ motion is likely to help maintain maximal absorption, retard colonic transit and, therefore, reduce stool frequency.

### Wireless motility capsule

After ingestion, this capsule, which is 26 mm by 13 mm in size, measures pH, pressure and temperature within the gastrointestinal tract. Data are wirelessly transmitted to a recorder worn externally. It is the only method that simultaneously records colonic pressure activity and transit^165^. In a large study, overall agreement between capsule and radiopaque marker measurements of colonic transit categorized as normal or slow was 87%^166,167^.

The ambulatory system enables studies to be completed at home. The capsule can reliably distinguish between normal and slow colonic transit. Colonic pressure activity is on average greater in constipation-predominant IBS than in functional constipation^168^, but there is considerable overlap between groups. Colonic pressure activity increases as the capsule moves distally through the colon^169^. As the location of the capsule in the colon is not monitored during its passage, the pressure cannot be attributed to a specific colonic region. Because pressures are recorded at only a single location, propagation of motor events cannot be assessed.

### Electromagnetic capsules

This equipment (3D-Transit; Motilis Medica SA) comprises a magnetic pill (a silicon-coated capsule, which is 21 mm by 9.8 mm in size, containing a permanent cylindrical magnet), a detection matrix (4 × 4 magnetic field sensors) that detects movements of the pill, and dedicated software. The matrix maps the location and movement of the pill within the colon by plotting the *x*, *y* and *z* coordinates and two inclination angles^170^.

The technique enables the calculation of both antegrade and retrograde movement and the speed of that movement in real time. By detecting pill rotation, the frequency of contractile events can be determined. Gastrointestinal transit times assessed by 3D-Transit capsules and standard radiopaque markers strongly correlate^171^. Currently, the use of this tool is limited to research studies. Colonic retention times have been evaluated in healthy adults^170–172^ and in patients with active ulcerative colitis^173^. All studies were conducted in Europe. The ambulatory system enables studies to be completed at home.

### Barostat

**Fig. 8:**
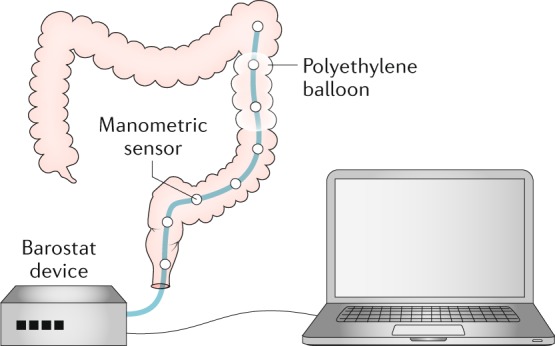
Assessment of colonic motility using a barostat. The barostat–manometric assembly is positioned in the descending colon with a polyethylene balloon in apposition to the colonic mucosa. The device maintains the balloon at a pressure that ensures that the colonic wall is not distended. Contractions of the colonic wall induce a decrease in the baseline balloon volume, which is recorded by the barostat.

The barostat was initially used to measure gastric^174,175^ and subsequently colonic motor function^118,176^. A highly compliant polyethylene balloon is placed into the cleansed colon using endoscopy and fluoroscopy. This balloon is connected to a barostat, which is a computer-controlled, motor-driven piston within a cylinder^92^ that can regulate and measure balloon pressure and volume. When the colon contracts against a balloon inflated to a fixed pressure, air is expelled from the balloon into the barostat. Conversely, when the colon relaxes, the barostat adds air to the balloon to maintain a constant pressure. Another option is to distend the balloon across a range of pressures and measure volume to derive pressure–volume relationships. Generally, a barostat balloon is combined with manometry pressure sensors^177^ (Fig. 8).

Unique to a barostat is its ability to detect colonic tone, nonlumen-occluding contractions, pressure–volume relationships, sensation and relaxation. Tone is crucial to colonic mixing and propulsion. In particular, the right colon has a propensity to relax. Baseline tone is a prerequisite for relaxation. Colonic distention to low pressure by a barostat does not alter motor activity recorded by manometric sensors. However, the possibility that a balloon distended to a low pressure might stimulate low threshold mucosal afferents cannot be excluded.

Eating increases tone to a lesser extent in the proximal colon than in the distal colon^178,179^. Eating also increases colonic phasic pressure activity. The response can begin within a few seconds after eating and generally lasts up to 1 h. Meal composition and caloric content are both important; a mixed meal containing >500 kcal predictably elicits a response^178,179^. Gastric distention and chemical stimulation by nutrients elicit comparable responses; lipids are the most potent stimuli, whereas amino acids have an inhibitory effect^180^. In addition to augmenting tonic and phasic motor activity, feeding also increases colonic sensation and affects recto–colonic reflexes^181^. This reflex might contribute to the postprandial urge to defecate and to postprandial abdominal discomfort in IBS.

When the colonic diameter exceeds 5.6 cm, a barostat is more sensitive than manometry for recording nonocclusive contractions^182^. A barostat can also record colonic relaxation, whereas manometry can only directly record contraction^176^. However, in contrast to manometry, a barostat cannot evaluate spatial patterns or propagation. Another aspect of the barostat is its ability to record colonic pressure–volume relationships and perception of distention^183,184^. Pressure–volume relationships are useful for assessing colonic biomechanical properties in health and disease and in response to pharmacological modulation.

### MRI

**Fig. 9:**
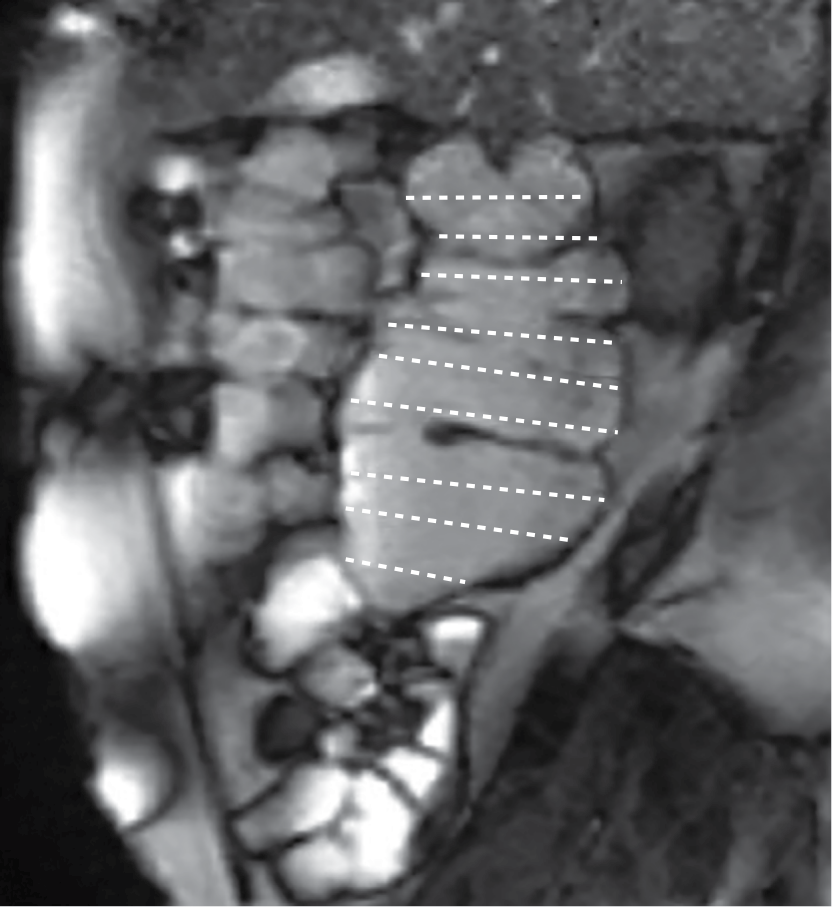
MRI assessment of colonic wall movement. MRI enables the measurement of various characteristics of the gastrointestinal tract, such as organ volumes, transit rate and motility. In this MRI of a sagittal section of the ascending colon, the horizontal lines define the colonic lumen. During cine MRI, changes in the lengths of the lines provide a measure of transverse wall velocity, which enables the calculation of a motility index (the percentage of data points at which the wall velocity is >0.5 mm/s).

MRI differs from other nonmanometric techniques as it does not require ionising radiation or invasive procedures, or the use of special contrast agents. It can be used to measure gastric, small intestinal and colonic volumes^185^, the physical chemical characteristics of the lumen environment^186^, transit rate, and to quantify motility, as reviewed elsewhere^187^. Cine imaging, or the ability to acquire multiple, temporally-resolved, images, enables the visual appreciation of wall movement; direct comparison with manometry showed good correlation between luminal occlusive contractions induced by bisacodyl and high amplitude propagated contractions^188^. Cine MRI can also readily detect the strong propulsive contractions induced by luminal distension with the osmotic laxative polyethylene glycol as fluid enters and distends the ascending colon. Thus, in the future, cine MRI could be relevant in the clinical assessment of constipation given that cine MRI requires only the ability to operate an MRI scanner, which is much more widely prevalent than expertise in manometry. Application in clinical practice is only just beginning, but increased motility after ingestion of polyethylene glycol has been demonstrated using a semiquantitative subjective assessment where motility was greater in healthy controls and patients with constipation-predominant IBS than in those with functional constipation^189^. A more objective method has also been developed, which confirmed the subjective assessments^189^, using a dual registration of abdominal motion (DRAM) technique to correct for respiratory artefacts. This correction is necessary when prolonged recordings are to be made (more than a single breath hold)^189,190^. Imaging the undistended colon using the contrasting signal between the colonic wall and its contents is possible but is less distinct and the anatomy is more variable, particularly in the sigmoid area compared with the distended colon. Hence, assessment that is more detailed requires further development of this technique. A very promising MRI tagging technique was reported in 2017 that enables observing movement of colonic contents^191^ (Fig. 9). This method shows axial flow of contents with minimal movements adjacent to the colonic wall, possibly reflecting impedance to flow by the haustra^191^. Although the length of MRI recordings in practice has no limit, the duration of recordings is often short (5–30 min) owing to a combination of expense and patient discomfort from close confinement in the scanner. This limitation makes this technique most suitable for studying short-lived provoked motility, for example after a laxative or pharmacological stimulant such as neostigmine or bisacodyl. Thus, at present, MRI should be seen as a complementary technique rather than as a substitute for manometry.

## Conclusions

In this Expert Consensus Statement, we report the results of the first translational consensus on colonic motility. To our knowledge, basic scientists and adult and paediatric gastroenterologists met for the first time with the aim of reviewing the current literature in conjunction with their own studies to find a common conceptual understanding of colonic motility and reach an agreement on terminology and the definitions of CMPs. The participants were selected based on their contribution to the current knowledge of these patterns in human and animal studies, both in vitro and in vivo. This effort was particularly useful, as it was immediately clear to all participants that concepts, terminology and definitions applied by basic scientists were frequently misinterpreted by clinicians and vice versa. The data presented in this manuscript represent the agreement reached by the consensus group with the main aim of furthering knowledge about colonic motility from a common understanding of the current state of the art, and to introduce uniformity in the different terminologies used in different laboratories (Table 4).

**Table 4:** Suggested terminology of colonic motor patterns

	Consensus term	Previous terms	Definition
Animal colon segments (in vitro)	Neural peristalsis	Long-distance contractions	Anterograde propulsive movements triggered by distension with liquids or solids; can be repetitive if stimulus persists
	Ripples	Ripples	Myogenic contractions generated by non-neuronal pacemaker cells; can be retrograde, anterograde or nonpropagating (chaotic)
	Colonic motor complex	Colonic migrating motor complexes; cyclic motor complexes; haustral progression; haustral boundary contractions	Neurogenic repetitive peaks of pressure and/or electrical activity; can be migrating or nonmigrating
	Neural retrograde propagating contraction	Retrograde propagating contraction	Neurogenic retrogradely propagating contractions
Human isolated colon (in vitro)	Repetitive motor pattern	Repetitive propagating sequences	Anterograde, retrograde or simultaneous pressure events triggered by enteric nerve stimulation or spontaneous
	Single motor pattern	Single propagating sequences	Anterograde or retrograde pressure events triggered by enteric nerve stimulation, balloon distension or spontaneous
Adult colonic recording (in vivo)	Simultaneous pressure increases	Simultaneous contractions; pan-colonic pressurizations; common cavity	Simultaneous pressure increases across multiple recording sites; may be associated with internal anal sphincter relaxation
	Haustral boundary pressure transients	Haustral boundary pressure transients (movement of haustra not conclusively demonstrated)	Rhythmic or continuous pressure increases that can occur in isolation at a single location or at spacings of 4–5 cm, moving across these sensors
	Intrahaustral activity	Intrahaustral segmental activity	Pressure waves propagating in both directions at ~3 cpm
	Cyclic propagating motor pattern	Segmental contraction; nonpropagating activity	Repetitive propagating pressure events with a frequency of 2–6 cpm; can propagate retrogradely, anterogradely or occur simultaneously across ≥2 channels
	Short single motor pattern	Low-amplitude propagating contraction	Isolated propagating contractions separated by intervals of >1 min; propagating anterogradely or retrogradely; typically spanning between 3 and 10 cm
	Long single motor pattern	Low-amplitude propagating contraction	Isolated propagating contractions separated by intervals of >1 min; propagating rapidly (>2 cm/s) primarily anterogradely for >30 cm
	Slow retrograde motor pattern	Not previously defined	A slow (<0.5 cm/s) retrogradely propagating contraction spanning >40 cm
	High-amplitude propagating contractions	High-propagating sequences; giant migrating contractions; high-amplitude propagating pressure waves	An array of pressure waves, with the majority having a trough-to-peak amplitude of >100 mmHg, that extend >20 cm along the colon
Paediatric colonic recording (in vivo)	Simultaneous pressure increases	Simultaneous contraction	Same as in adults
	Haustral boundary pressure transients	Not previously defined	Not previously defined
	Intrahaustral activity	Not previously defined	Not previously defined
	Cyclic propagating motor pattern	Segmental contraction; nonpropagating activity	Same as in adults
	Short single motor pattern	Low-amplitude propagating contraction	Same as in adults
	Long single motor pattern	Low-amplitude propagating contraction	Same as in adults
	Slow retrograde motor pattern	Not previously defined	Same as in adults
	High-amplitude propagating contractions	High-propagating sequences	Same as in adults

Definitions of in vivo recordings are based on high-resolution manometry. cpm, cycles per minute.

The introduction of colonic HRM has enabled us to redefine knowledge of the normal CMPs in humans. In particular, it has enabled us to better identify low-amplitude activity and to obtain simultaneous recordings of both colonic and anorectal motor activity, which was previously not possible owing to the thickness of the required catheter and the large distance between recording sensors. HRM has demonstrated that the colonic pressurizations or simultaneous pressure increases, the retrograde cyclic activity and the intrahaustral activity are important and common CMPs in adult humans.

The comparison of data on animal and human motor patterns was an important aspect of the consensus meeting (Table 5). Despite the very different experimental conditions used in animal and human studies, similarities in the cellular mechanisms are sufficient to expect similarities in the motor patterns. Further research is needed to conclusively determine which human motor patterns might correspond to the motor patterns described in experimental animals^190^. The motor patterns in animals summarized in this article include myogenic ripples, myogenic high-frequency ripples, myogenic slow phasic contractions, neurogenic colonic motor complexes and neural peristalsis.

**Table 5:** Possible correspondence between colonic motor patterns in different settings

Animal colon segments (in vitro)	Human isolated colon (in vitro)	Adult colonic recording (in vivo)	Paediatric colonic recording (in vivo)
Neural peristalsis	Not yet studied	High-amplitude propagating contractions; long or short single motor pattern	High-amplitude propagating contraction; long or short single motor pattern
Ripples	Not yet studied	Cyclic propagating motor pattern; intrahaustral activity	Cyclic propagating motor pattern
Colonic motor complex	Repetitive motor pattern	Simultaneous pressure increases	Simultaneous pressure increases
Colonic migrating motor complex	Not yet studied	Haustral boundary pressure transients	Not observed
Neural retrograde propagating contraction	Not observed	Slow retrograde motor pattern	Not observed

Isolated human colonic strips do not contain the entire neural circuits to generate complex motor patterns, but are useful to understand myogenic mechanisms, such as ripples associated with slow wave activity and slow phasic contractions and their modulation by neural inputs.

The most obvious similarity is that between neural peristalsis that propels the contents in animals when distended and the HAPCs and long single motor patterns in humans. In both the animal bowel and the human bowel, these motor patterns are dependent on the activation of enteric neural circuits by the bowel contents with greater or lesser dependence from extrinsic excitatory neural inputs. Defecation in omnivores is sensitive to environmental conditions, whereas pellet excretion in herbivores is clearly quasi-continuous^192–195^.

Less clear is the homology between the various types and descriptions of CMCs in experimental animals and similar patterns in humans. In animals, the CMC seems to originate from the interaction between neurogenic excitatory activity that occurs at intervals close to 1 min and the myogenic ripples. In the proximal colon of herbivores, the slow propagation of haustral boundaries that facilitates the formation of faecal pellets is driven by neurogenic, slowly propagating CMCs. Neurogenic slow propagation of haustrations might also occur in humans but has not yet been convincingly demonstrated. The neurogenic CMC elicited by continuous distension of the animal colon^42,81^ might correspond to the simultaneous and quasi-simultaneous motor complexes described in the isolated human colon in vitro^86,87^ and to the repetitive pan-colonic simultaneous events (pressurizations) described in humans in vivo that occur at a frequency of ~1 per minute^7,8,196^.

The widely described ripples in the colon of animals, generated by slow waves, have also been identified in vitro in the human colon where they generate regular contractions at 2–6 cpm, and are termed intermediate-frequency contractions. In animals, the ripples are generated by the pacemaker system located at the submucous side of the circular muscle (ICC-SMP). In humans, these ripples are present in vivo throughout the colon and seem to be part of the cyclic propagating motor pattern. HRM recordings from the human sigmoid colon have demonstrated a retrograde propagation of the cyclic motor activity, perhaps indicating a mechanism that prevents premature rectal filling^197^. Extrinsic excitatory neural inputs observed after feeding can increase the amplitudes of the colonic ripples, which might then become mechanically more effective^6^. The intrasegmental activity reported in humans^8^ as pressure waves propagating in both directions at ~3 cpm is also likely to represent myogenic ripples.

No clear evidence exists from human colonic manometry studies regarding the occurrence of the high-frequency ripples that are commonly observed in animals, although a similar underlying pacemaker system (the ICC-MP) exists in humans that generates the electrical myenteric potential oscillations at a higher frequency than that of the slow waves. Slow phasic myogenic contractions might correspond to the low-frequency contractions described in human samples in vitro and have been observed in isolated preparations from animals. In summary, a number of similarities exist between motor patterns described in animals and those in the human colon, both in vitro and in vivo.

The review and discussion concerning the CMPs recorded in vivo in children has been particularly illuminating. The findings from HRM studies on what should be considered normal in healthy adults have raised questions regarding the manometric criteria in protocols for surgical intervention in children with refractory constipation. Healthy children cannot be studied for ethical reasons. Moreover, the characteristics of propagating CMCs that lead to mass movements, notably HAPCs, are regarded as key features in paediatric clinical decision-making because some studies in children suggest that the characteristics of HAPCs can predict the response to treatment. However, these characteristics are not considered in the decision making for adults. The sole focus on HAPCs will probably be replaced by a more detailed assessment, including the identification and quantification of other motor patterns and differential assessment of regional motor activity. Thus, we clearly need to further expand our knowledge about normal motor patterns and ultimately establish a uniform protocol by which to perform and analyse colonic motility studies in both children and adults. This harmonization of protocols would enable the pooling of multicentre results and modification of treatment outcomes.

The review of the literature regarding the use of nonmanometric techniques indicates that colonic scintigraphy has the potential to clarify the inter-relationship between the various patterns of motility and the transit of digesta, and that barostatic studies have the ability to quantify colonic tone and wall compliance. However, scintigraphy involves exposure to radiation and the barostat can assess activity only over a localized area. The wireless motility, pH and pressure capsule (SmartPill) has the disadvantage that it cannot assess the propagation of motor events and, as the location of the capsule in the colon is unknown, the pressure registration cannot be attributed to a specific colonic region. Promising techniques seem to be the 3D-Transit electromagnetic capsules that can assess the movements of colonic contents, and MRI that can measure different colonic and noncolonic parameters simultaneously (gastric, small intestinal and colonic volumes, the physical and chemical environment, transit and motility).

The current literature regarding colonic motility in humans has many deficiencies. In general, the studies applying colonic manometry included low numbers of individuals and often highly selected patients. Future manometric studies in larger populations of healthy individuals are needed to confirm the findings with HRM and to establish more reliable normal ranges. Combined manometric and transit studies could help to determine the functional role of particular motor patterns. Future research should also focus on the possible mechanisms that control these patterns and elucidate possible pharmacological strategies for therapeutic modulation. Further studies, ideally multicentre studies, are needed to re-evaluate the utility of colonic manometry in decision making in clinical practice. For example, in patients with chronic constipation who have failed to respond to current treatments, HRM could give a more complete description of motor patterns to subtype constipation, enabling treatment around that subtyping^198^.

Medical and surgical decisions for children are currently based on colonic manometry studies, but no studies have conclusively demonstrated that characterizing and quantifying CMPs improves long-term outcome. This aim could be achieved only if colonic manometric procedures are standardized between centres. Adult and paediatric gastroenterologists who perform such procedures and scientists with expertise in colonic physiology should work together to achieve such standardization. Both in children and adults, the relevance of poorly propagating HAPCs in disorders of motility needs to be clarified. A total absence of propagated contractions in response to colonic stimulants can be considered a reliable indicator of colonic inertia, but the clinical significance of HAPCs that propagate only partially and are followed by simultaneous increases in pressure is less clear (Fig. 6b). Do simultaneous increases result from a ‘common cavity’ phenomenon or do they represent true simultaneous contractile activity? Unlike a contractile event, a common cavity was hypothesized to occur when a propagating contraction in the proximal regions of the colon moves content (gaseous, liquid or solid) into the distal regions. The propagating contraction ends and, if the anal sphincters remain closed, the content moving ahead of the propulsive motor pattern causes a simultaneous rise in pressure throughout the distal regions of the colon. Is the consistent failure of the colonic segment to develop propagated contractions the primary cause (and is the disorder, therefore, amenable to surgical resection) or is it a secondary effect of the patient’s severe constipation? Finally, the clinical relevance of the CMPs identified by HRM in children needs to be assessed.
